# Vitamin D3 Improves Hypothalamic–Pituitary–Adrenal Axis Function, Immunological Responses, and Gut Dysbiosis in Sleep Desynchrony

**DOI:** 10.1002/brb3.71084

**Published:** 2025-11-21

**Authors:** Changwei W. Wu, Yen‐Ju Huang, Jin‐Wei Xu, Hsien‐Yu Fan, Jun‐Lan Zeng, Yu‐Chen S. H. Yang, Yu‐Tang Tung

**Affiliations:** ^1^ Graduate Institute of Mind Brain and Consciousness Taipei Medical University Taipei Taiwan; ^2^ Research Center of Sleep Medicine Taipei Medical University Hospital Taipei Medical University Taipei Taiwan; ^3^ Department of Pathology Ditmanson Medical Foundation Chia‐Yi Christian Hospital Chiayi Taiwan; ^4^ Department of Forestry National Chung Hsing University Taichung Taiwan; ^5^ School of Nutrition and Health Sciences College of Nutrition Taipei Medical University Taipei Taiwan; ^6^ Graduate Institute of Biotechnology National Chung Hsing University Taichung Taiwan; ^7^ Joint Biobank Office of Human Research Taipei Medical University Taipei Taiwan; ^8^ Graduate Institute of Metabolism and Obesity Sciences College of Nutrition Taipei Medical University Taipei Taiwan; ^9^ Advanced Plant and Food Crop Biotechnology Center National Chung Hsing University Taichung Taiwan; ^10^ Cell Physiology and Molecular Image Research Center Wan Fang Hospital Taipei Medical University Taipei Taiwan; ^11^ Department of Pharmacology College of Medicine Kaohsiung Medical University Kaohsiung Taiwan

**Keywords:** chronic sleep desynchrony, gut dysbiosis, hypothalamic–pituitary–adrenal (HPA) axis, short chain fatty acid, vitamin D3

## Abstract

**Purpose:**

Chronic sleep desynchrony proliferates in modern society, characterized by extended wakefulness and disrupted circadian rhythms over a long period, and is associated with various physiological and immune dysfunctions. This study investigated the health effects of long‐term sleep desynchrony and subsequent vitamin D3 treatment, alongside sleep recovery, on biological factors in C57BL/6J mice by assessing their body weight, metabolic activity, stress response, immune function, and gut microbiota composition.

**Methods:**

Mice underwent 28 days of sleep desynchrony using a Lafayette chamber to model chronic disruption of the HPA axis, immune function, and gut microbiota.

**Finding:**

Sleep desynchrony did not significantly alter body weight, food intake, or water consumption, suggesting that metabolic homeostasis was preserved. However, stress hormone analysis revealed impaired HPA axis regulation in the sleep‐desynchronized mice, as indicated by suppressed corticosterone levels after dexamethasone injection. Notably, vitamin D3 treatment restored the HPA axis function by enhancing corticosterone suppression. Behavioral tests of the mice indicated that sleep desynchrony reduced nest‐building ability, locomotor activity, and anxiety‐like behaviors, whereas vitamin D3 treatment improved their motor function. Furthermore, vitamin D3 treatment alleviated immune dysregulation by reducing neutrophil levels and increasing lymphocyte counts, suggesting a role in restoring immune balance after sleep desynchrony. Regarding gut microbiota composition, sleep desynchrony disrupted microbial diversity, reducing Bacteroidota and increasing Firmicutes, leading to dysbiosis. Vitamin D3 treatment shifted the microbiota composition toward eubiosis, aligning it closer to control‐group levels. Additionally, sleep desynchrony impaired the expression of tight junction proteins in the proximal colon, compromising intestinal barrier integrity. Vitamin D3 treatment restored the levels of key tight junction proteins, such as ZO‐1 and claudin, thereby maintaining gut barrier function.

**Conclusion:**

Vitamin D3 treatment serves as a promising intervention for mitigating the adverse health effects of chronic sleep desynchrony on the HPA axis, immune function, and gut microbiota, providing potential therapeutic benefits for preserving physiological homeostasis in the middle of a long‐term sleep deficiency or chronic sleep deprivation.

## Graphical abstract

This study examined the effects of chronic sleep desynchrony and vitamin D3 supplementation on stress responses, immune function, intestinal barrier integrity, and gut microbiota in mice. This work demonstrates that vitamin D3 supplementation can partially restore HPA axis responsiveness, rebalance immune cell profiles, and ameliorate gut dysbiosis, thereby offering translational relevance for mitigating the multifaceted health risks associated with shift work.

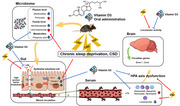

## 1 Introduction

Shift work plays a crucial role in the modern working environment. It supports sustainability, customer service, public safety, and health care, and offers economic advantages. This type of work is essential in sectors such as health care, transportation, industry, commerce, and hospitality (Kalkanis et al. 2023). Currently, approximately 20% of the global workforce is engaged in shift work. Statistical analyses have revealed a 15% increase in the prevalence of shift work across several European countries. In the United States, the incidence of shift work has reached 20% among men and 11% among women (Soltanzadeh et al. 2024). Notably, shift work has several psychophysiological effects that are primarily related to circadian misalignments and sleep disturbances, and shift‐work sleep disorder is estimated to affect 10% to 38% of the workforce (Sateia 2014; Pallesen et al. 2021). The effects are particularly strong for individuals working rotating night shifts, for whom sleep is often prematurely interrupted because of the influence of circadian rhythms, leading to severe sleepiness and reduced performance capacity (Åkerstedt 1990).

Sleep is regulated by the two‐process model, in which the homeostatic process reflects the accumulation of sleep pressure during wakefulness and the circadian process follows a 24‐h rhythm aligned with the light–dark cycle. Shift workers often experience irregular sleep schedules that cause misalignments between the two processes, leading to chronic sleep deprivation (CSD), impaired sleep quality, insomnia, and excessive daytime sleepiness, all of which contribute to decreased alertness and poorer cognitive performance (Drake et al. 2004). Sleep desynchrony considerably affects the mental health of shift workers. Studies have reported that shift workers are twice as likely as day‐only workers to report depressive symptoms (Driesen et al. 2010). Furthermore, shift workers have a 1.5‐fold greater risk of experiencing severe depressive symptoms (Lee et al. 2016). Although some studies have identified an increased risk of anxiety and depression among shift workers (Lin et al. 2012; Bara and Arber 2009), others have indicated better psychological well‐being among shift workers compared with non‐shift workers (Bildt and Michélsen 2002; Nabe‐Nielsen et al. 2011). These conflicting findings may reflect variations in the contexts, occupational settings, and population characteristics of the aforementioned studies. In addition to mental health problems, shift workers are at a higher risk of experiencing other health conditions, such as metabolic syndrome (De Bacquer et al. 2009; Pietroiusti et al. 2010), cardiovascular diseases (particularly coronary heart disease) (Torquati et al. 2018), and gastrointestinal disorders (Drake et al. 2004; Knutsson and Bøggild 2010). Therefore, employing effective interventions to mitigate these health risks is crucial. Research has indicated that sufficient high‐quality sleep after night shifts can reduce fatigue and enhance recovery, particularly among nurses (Silva‐Costa et al. 2011; Konya et al. 2022). Thus, for shift workers, implementing targeted interventions may help mitigate the adverse health effects of sleep desynchrony and improve their quality of life and cognitive performance.

Vitamin D supplementation has been explored as a potential intervention for enhancing sleep quality. Majid et al. (2018) reported that 8 weeks of vitamin D supplementation reduced sleep latency and increased sleep duration in middle‐aged individuals. Furthermore, dietary vitamin D intake was demonstrated to significantly reduce the risk of anxiety and sleep disorders, although no significant association between dietary vitamin D intake and depression was identified (Arabshahi et al. 2024). Shift work was also reported to be linked to lower vitamin D levels, more depressive symptoms, and poorer sleep quality (Park et al. 2019). A study suggested that sleep deprivation can have lethal effects in model organisms, with these effects primarily resulting from the accumulation of reactive oxygen species (ROS), particularly in the gut (Vaccaro et al. 2020). Because of its ability to reduce anxiety, alleviate sleep disorders, and act as an antioxidant to counter ROS accumulation, the current study selected vitamin D for the experimental treatment following sleep desynchrony. Moreover, vitamin D has been shown to modulate gut barrier integrity and intestinal inflammation through cytokine regulation and microbiota interaction, highlighting its potential in gastrointestinal and systemic immune regulation (Vernia et al. 2022). While the individual roles of vitamin D3 in modulating immune responses, regulating stress pathways, and influencing gut microbiota have been reported, no study to date has comprehensively evaluated these systems in an integrated manner under conditions of chronic sleep desynchrony. Therefore, this study investigated the effects of continuous sleep desynchrony and subsequent interventions, including vitamin D3 treatment and sleep recovery, on the body weight, metabolic activity, stress response, immune function, and gut microbiota composition in mice.

## 2 Materials and Methods

### 2.1 Animals and Procedures

This study obtained 5‐week‐old male C57BL/6J mice from the National Laboratory Animal Center in Taipei, Taiwan. The mice were housed in the Laboratory Animal Center of National Chung Hsing University under controlled environmental conditions, specifically, a 12‐h light–dark cycle, a stable temperature of 23°C ± 2°C, and relative humidity maintained at 60% ± 10%. The mice were given unrestricted access to water and fed a standard laboratory diet. All procedures involving the animals were reviewed and approved by the Institutional Animal Care and Use Committee of National Chung Hsing University (approval number: 111‐004R). Prior to the sleep deprivation experiment, the mice were acclimatized to their environment and diet for 1 week.

The present study involved a total of 24 mice, which were randomly divided into four groups (*n* = 6 per group): normal sleep (NS), sleep recovery after deprivation (CSDR), and CSD combined with 1000 IU/kg of vitamin D3 supplementation (CSDVD). The CSD protocol was applied for the CSD, CSDR, and CSDVD groups for 28 days. Specifically, the CSDR group had additional 7‐day recovery period without applying the CSD protocol, mimicking the “back to normal” period after exposing to chronic shift work or chronic sleep deficiency in human. The vitamin D3 dosage of 1000 IU/kg was selected based on previous rodent studies demonstrating neuroimmune and behavioral modulation at this dose without toxicity (Park et al. 2020; Seldeen et al. 2020; Tan et al. 2021). This dose is considered physiologically relevant for mice and has been widely used in preclinical investigations of vitamin D3 function. Throughout the experiment, the mice's body weight and food intake were monitored regularly. At the end of the experimental period, fecal samples were collected, and behavioral tests were conducted. To minimize experimental bias, behavioral assessments and sample analyses were performed by investigators who were blinded to the group allocations. Upon the completion of the experiment, all mice were euthanized, and blood and tissue samples were collected for further analysis. Blood samples were drawn through retro‐orbital bleeding using heparinized capillary tubes. A 300‐µL sample of whole blood was used for white blood cell analysis, which was conducted at Accuspeedy Medical Laboratory in Tainan, Taiwan. Additionally, brain tissues from the hypothalamus, as well as samples from the proximal colon, were collected for subsequent examination.

## 3 Protocol of CSD

CSD was induced for 28 days using a sleep fragmentation device (Lafayette Instrument Company, Lafayette, IN, USA), following a protocol adapted from Cabrera‐Aguilera et al. (2020) with minor modifications. The device comprised a nearly silent mechanical motor that moved a horizontal bar across the floor of a standard mouse cage. This bar gently swept back and forth just above the cage floor to disrupt sleep while ensuring minimal noise and stress for the animals. The automated system could be operated without the need for human interaction, further reducing potential stress. The bar was programmed to sweep once per minute during the mice's designated sleep period (8 a.m. to 6 p.m., 10 h in total), and this protocol was applied continuously for 28 days.

## 4 Dexamethasone Suppression Test and Corticosterone Level

Dexamethasone, an exogenous steroid, suppresses the secretion of adrenocorticotropic hormone by activating negative feedback on the pituitary gland, which renders it a useful tool for evaluating the function of the hypothalamic–pituitary–adrenal (HPA) axis (Pariante and Lightman 2008). In the present experiment, corticosterone levels were measured following intraperitoneal injections of dexamethasone to assess HPA axis activity.

On Day 18, the mice received an intraperitoneal injection of saline, and plasma samples (100 µL) were collected from the cheek after 1 h, with these samples serving as the baseline (blank) group. The following day (Day 19), the same mice were injected intraperitoneally with dexamethasone, and after 1 h, plasma samples (100 µL) were collected again, with these samples representing the test group. Corticosterone suppression efficiency was calculated using the formula: 100 − ([corticosterone level] after dexamethasone injection/[corticosterone level] after saline injection) × 100.

At the conclusion of the experiment, the corticosterone levels of the plasma samples were quantified using the Corticosterone AssayMax enzyme‐linked immunosorbent assay (ELISA) Kit (Assaypro, Saint Charles, MO, USA).

## 5 Behavioral Test

### 5.1 Nest‐Building Test

The nest‐building test (NBT) can be used to assess the health status of mice because they tend to exhibit reduced nest‐building behavior when they experience pain or stress (Jirkof 2014). Before the test, each mouse was housed individually, and each cage was supplied with six cotton rolls (ARBOCEL Roll, USA) as nesting material. The test began at the onset of the light–dark cycle and lasted for 12 h. After the test, the nest‐building results were photographed, and nest quality was scored in accordance with the criteria outlined by Obermueller et al. (2021). Nest quality was scored from 0 to 10 on the basis of several factors, such as the amount of cotton used, the presence of a nest shape, and the nest height, all of which reflect the health condition of mice.

### 5.2 Open Field Test

The open field test (OFT) was conducted in accordance with the protocol outlined by Seibenhener and Wooten (2015). This assessment was performed after Day 27 of CSD (NS, CSD, and CSDVD groups) and after Day 34 for the CSDR group. Each mouse was placed individually in a transparent acrylic enclosure measuring 45 cm × 45 cm × 40 cm, with a central area of 30 cm × 30 cm, and the mice were allowed to explore the space for 15 min. To evaluate anxiety‐related behavior and locomotor activity, the number of times the mice entered the central area during the first 5 min and the total distance they covered over the entire 15‐min period were automatically recorded using the software TopScan Lite (TopScan Lite, CleverSys, USA). The apparatus was thoroughly cleaned with 75% ethanol between trials to prevent any influence from prior animals.

### 5.3 Elevated Plus Maze

The elevated plus maze (EPM) test was conducted in accordance with the protocol established by Walf and Frye (2007) and was performed after Day 27 of NS, CSD, and CSDVD groups/Day 34 for the CSDR group. The EPM apparatus used in this assessment comprised two open arms (35 cm × 5 cm) and two closed arms (35 cm × 5 cm) with 20‐cm‐high walls, all connected by a central platform elevated 40 cm above the ground. Each mouse was placed at the center of the maze, facing one of the open arms, and allowed to explore for a 10‐min session. The entire session was recorded for subsequent analysis. The number of times a mouse entered the open arms (open/open + closed) or the closed arms (closed/open + closed) was automatically tracked and analyzed using the software CleverSys. The apparatus was thoroughly cleaned with 75% ethanol between trials to prevent any influence from prior animals.

## 6 RNA Extraction and Real‐Time Quantitative PCR (qPCR)

RNA samples were extracted from various brain regions, including the prefrontal cortex (PFC), hippocampus, hypothalamus, bed nucleus of the stria terminalis, and cerebral cortex, and preserved in RNA Save (Biological Industries, Kibbutz Beit Haemek, Israel) before being stored at −80°C for subsequent analysis. Total RNA extraction was conducted using the EZ‐RNA II total RNA isolation kit (Biological Industries). After extraction, 1 µg of RNA in a 20‐µL reaction mixture was reverse transcribed into cDNA using the ToolsQuant II Fast RT Kit (TOOLS, Taipei, Taiwan). Quantitative PCR (qPCR) was subsequently performed using the TOOLS 2X SYBR qPCR Mix (TOOLS) and analyzed with a LightCycler 480 system (Roche, Basel, Switzerland). The resulting cDNA was used to measure the expression of key gene categories, including inflammatory genes (*TNF‐α*, *IL‐6*, and *IFN‐γ*) and circadian genes (*Bmal‐1*, *Clock*, *Cry1*, *Cry2*, and *Per2*). The relative mRNA expression levels of each target gene were quantified on the basis of threshold cycle values and normalized to the expression of glyceraldehyde‐3‐phosphate dehydrogenase.

## 7 Flow Cytometry Immunoassay

All antibodies used in the present study were derived from naive CD4^+^ T cells isolated from mouse spleens in accordance with the protocol outlined by Yang et al. (2018). Spleen tissues from each experimental mouse were minced into small fragments and mechanically disrupted to release cells. A single‐cell suspension was prepared by filtering the disrupted tissue through 200‐mesh screens. The procedures for labeling, permeabilization, and fixation were performed in accordance with the relevant kit instructions. The antibodies used comprised MS FITC Rat anti‐mouse CD4 (Clone GK1.5, Biolegend, San Diego, CA, USA), MS PE anti‐mouse NK 1.1 (Clone PK136, Biolegend), MS PE anti‐mouse IFN‐γ (Clone XMG1.2, Biolegend), and MS PE anti‐mouse IL‐4 (Clone PC61, BD Biosciences, San Jose, CA, USA). Flow cytometric analysis was conducted using a Beckman Coulter FC500 cytometer (Beckman Coulter, Brea, CA, USA), with data analysis performed using the software CXP.

## 8 Western‐Blot Analysis

Protein expression levels in the proximal colon were analyzed using Western blotting, in accordance with the method described by Ho et al. (2019). Membranes were incubated with primary antibodies targeting ZO‐1, occludin, claudin, and β‐actin at 4°C for 12 h on a shaker. The primary antibodies used in the present study comprised anti‐ZO‐1 (Cat# 21773‐1‐AP, 1:1000, Proteintech), anti‐occludin (Cat# 27260, 1:1000, Proteintech), anti‐Claudin (Cat# AF0127, 1:1000, Affinity), and anti‐β‐actin (Cat# 4970S, 1:3000, Cell Signaling). Immunoreactive bands were detected using enhanced chemiluminescence, and protein expression levels were quantified through densitometry by using the software ImageJ (Wayne Rasband, Madison, WI, USA). The results were normalized to β‐actin levels and expressed as fold changes relative to the control group.

## 9 Fecal Microbiota Analysis

The amplification and library preparation of the 16S rRNA gene were performed in accordance with Illumina's recommended protocols (https://support.illumina.com/downloads/16s_metagenomic_sequencing_library_preparation.html). Specifically, the universal primers 341F (forward primer; 5'‐CCTACgggNggCWgCAg‐3') and 805R (reverse primer; 5'‐gACTACHCggg‐TATCTAATCC‐3'), which include Illumina overhang adapter sequences, were used to amplify the V3–V4 region of the bacterial 16S rRNA gene through PCR with a limited number of cycles. After amplification, the Nextera XT Index Kit (Illumina, San Diego, CA, USA) was employed to add Illumina sequencing adapters and dual‐index barcodes to the resulting amplicons. The quality and quantity of the constructed library were evaluated using a Qsep100 Analyzer (BiOptic, Taipei, Taiwan). For additional details on the methodology, including database use, validation, and data analysis, refer to Tung et al. (2021). Functional profiling was conducted with PICRUSt2 (v2.3.0_b), which predicts gene family and pathway abundances (e.g., KEGG Orthologs and MetaCyc pathways) from phylogenetic placement of ASVs. Differential abundance analyses of predicted functions were carried out using ALDEx2 (v1.18) in R (v 4.5.1), applying centered log‑ratio (clr) transformation and Monte Carlo sampling to account for compositionality. Group comparisons were assessed using Welch's t‐test and Wilcoxon rank‐sum test, with *p* values corrected by Benjamini–Hochberg false discovery rate (FDR) correction. Beta diversity was assessed using weighted UniFrac and generalized UniFrac (α = 0.5) distance metrics. Principal coordinate analysis (PCoA) was used for visualization. Group differences were statistically tested using PERMANOVA via the adonis function (vegan v 2.6‐4) in R (v 4.5.1).

## 10 Endotoxin Markers in Plasma

The endotoxin marker LPS was detected using a ToxinSensor Chromogenic LAL Endotoxin Assay Kit (Cat# L00350, GenScript, Piscataway, NJ). ELISA procedures were performed in accordance with the manufacturer's instructions.

## 11 Fecal Short Chain Fatty Acid Analysis

Fecal samples weighing 40 mg were collected and immediately stored in 400 µL of water containing 0.5% phosphoric acid. After collection, the samples were rapidly frozen at −80°C. Prior to analysis, the samples were thawed, homogenized, and centrifuged at 14,000 rpm for 15 min, after which the supernatant was carefully collected. An equal volume of ethyl acetate was subsequently added to the supernatant, followed by vigorous vortexing for 2 min. The mixture was centrifuged again at 14,000 rpm for 15 min, and the organic phase was isolated and stored at −80°C until further analysis. Gas chromatography was subsequently performed in accordance with the protocol established by Tung et al. (2021). Short chain fatty acids (SCFAs) were identified by comparing the recorded retention times with those of established standards, including those for acetic acid, propionic acid, and butanoic acid.

## 12 Statistical Analysis

Statistical analysis was conducted using the software GraphPad Prism (Version 6, GraphPad Software, San Diego, CA, USA), with the data expressed as means ± standard errors of the mean (SEMs). Data were analyzed by performing the Mann–Whitney U test, a nonparametric statistical test. *p* values of <0.05 were regarded as indicative of significant differences.

## 13 Results

### 13.1 Effects of Vitamin D3 Supplementation and Sleep Recovery on Body Weight, Food Intake, and Water Intake in Mice With Sleep Desynchrony

As presented in Figure S1A, during the course of the experiment, the body weights of the mice in the NS, CSD, CSDR, and CSDVD groups exhibited normal and gradual increases, and no significant differences between the initial and final body weights were identified in the NS, CSD, CSDR, or CSDVD groups. Notably, no significant differences in food intake (Figure S1B) or water intake (Figure S1C) were identified among the groups, indicating that 28 days of sleep desynchrony did not significantly affect the body weight, food intake, or water intake of the mice.

### 13.2 Effects of Vitamin D3 Supplementation and Sleep Recovery on Stress Hormones in Mice With Sleep Desynchrony

**FIGURE 1:**
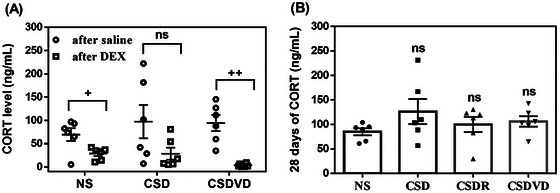
Effects of vitamin D3 treatment and sleep recovery on (A) dexamethasone suppression test results on Day 19 and (B) corticosterone levels on Day 28. NS: normal sleep group; CSD: chronic sleep deprivation group; CSDR: CSD followed by sleep recovery; CSDVD: CSD combined with vitamin D3 supplementation. Values are presented as means ± SEMs (*n* = 6). The unpaired one‐sided U test was performed for statistical analysis. ^+^ *p* < 0.05 and ^++^ *p* < 0.01 compared with the corticosterone level before dexamethasone suppression. ns indicates no statistical significance.

Dexamethasone is used to assess the function of the HPA axis, which regulates stress responses and corticosterone secretion. The NS group exhibited a significant decrease in corticosterone levels after dexamethasone injection, indicating a normal HPA axis suppression response (Figure 1A). By contrast, the CSD group did not exhibit a significant decrease in corticosterone levels after dexamethasone injection (Figure 1A), suggesting HPA axis dysfunction due to sleep desynchrony. When the CSDVD group underwent vitamin D3 treatment, dexamethasone significantly suppressed corticosterone secretion (Figure 1A). These findings highlight that vitamin D3 supplementation helps to ameliorate HPA axis dysfunction caused by sleep desynchrony, thereby restoring the dexamethasone suppression effect and enhancing corticosterone inhibition. In addition, the corticosterone levels of the NS, CSD, and CSDVD groups did not differ significantly throughout the 28 days of sleep desynchrony (Figure 1B). This suggests that, despite the presence of chronic sleep desynchrony, corticosterone levels remained stable across all groups.

### 13.3 Effects of Vitamin D3 Supplementation on Anxiety and Locomotor Activity in Mice With Sleep Desynchrony

**FIGURE 2:**
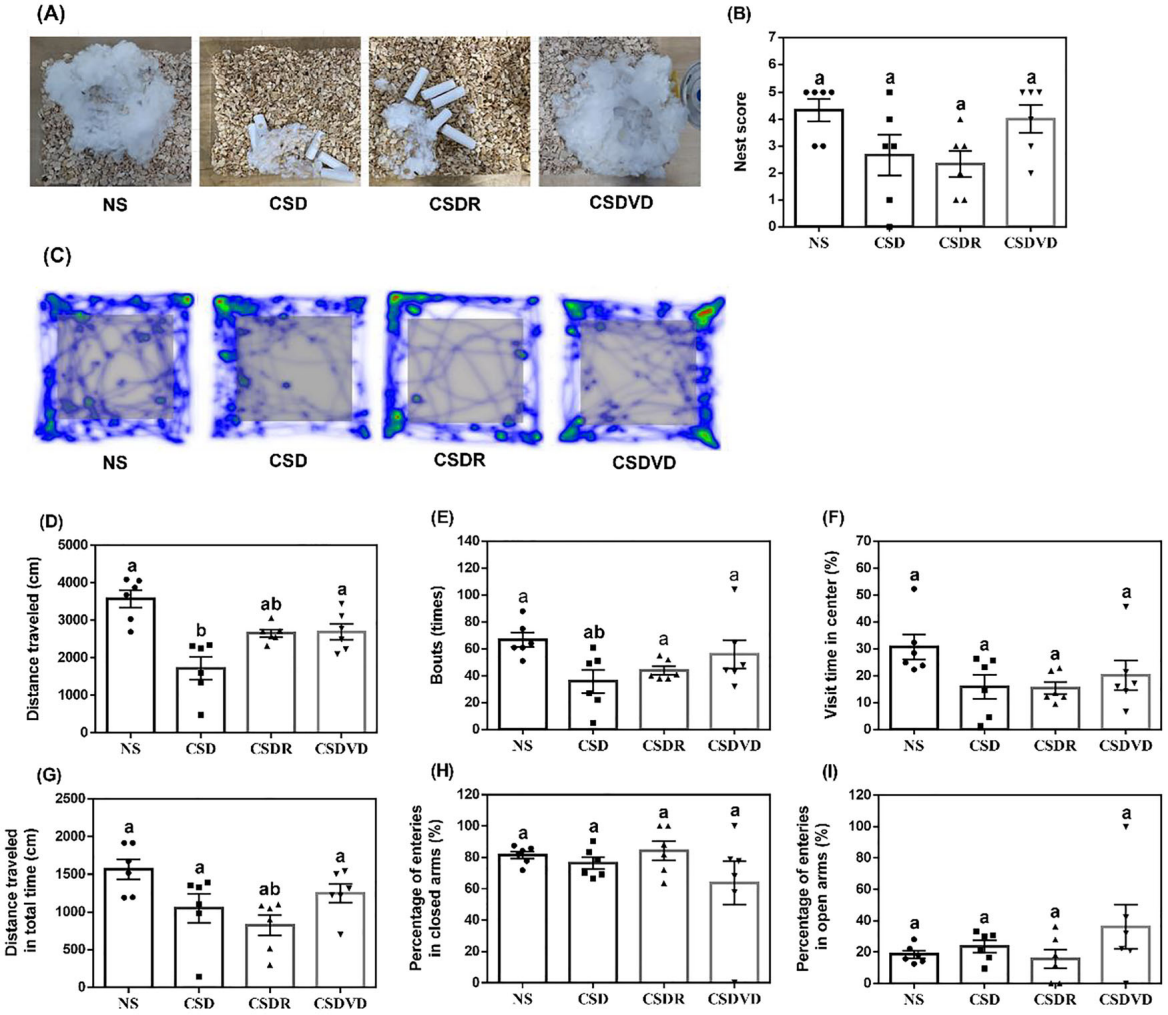
Effects of vitamin D3 treatment and sleep recovery on anxiety and locomotor activity in mice with sleep desynchrony. (A) Representative images from nest‐building test. (B) Nest scores from nest‐building test. (C) Representative images from open field test. (D) Results from open field test for total distance traveled. (E) Results from open field test for number of entries into center. (F) Results from open field test for spending time of visits into center. (G) Results from elevated plus maze test for total distance traveled. (H) Percentage of entries made into closed arm of elevated plus maze. (I) Percentage of entries into open arm of elevated plus maze. NS: normal sleep group; CSD: chronic sleep deprivation group; CSDR: CSD followed by sleep recovery; CSDVD: CSD combined with vitamin D3 supplementation. Values are presented as means ± SEMs (*n* = 6). The unpaired one‐sided U test was performed for statistical analysis. #*p* < 0.05 and ##*p* < 0.01 compared with the NS group. *p < 0.05 and **p < 0.01 compared with the CSD group. ns indicates no statistical significance.

The NBT is used to assess the cognitive function, motivation, physical health, and emotional well‐being of mice, rendering it a useful tool for studying depression, neurodegenerative diseases, and other mental health conditions in such animals. Figure 2A presents representative images of the nests built by the mice in this study, revealing that the NS and CSDVD groups built higher‐quality nests with better performance in completeness and structure relative to those of the CSD and CSDR groups. As presented in Figure 2B, the NS group achieved the highest nest score, whereas the CSD group exhibited a slight decline in nest‐building ability. However, the CSDVD group exhibited slight improvements relative to the CSD group.

The OFT provides a general assessment of locomotor activity and anxiety‐like behaviors, with a focus on activity levels and exploration patterns. As presented in Figure 2C, the mice in the NS group were more active in the test arena, whereas those in the CSD group exhibited a substantial reduction in movement range. The mice in the CSDR and CSDVD groups exhibited improvements in their movement range. The results for distance traveled indicated that compared with the mice in the NS group, those in the CSD group exhibited a significant reduction in distance traveled, suggesting that sleep desynchrony reduced their activity levels (Figure 2D). The mice in the CSDR and CSDVD groups achieved increased travel distances (Figure 2D). As indicated in Figure 2E,F, the NS group had the highest number of entries into the center and the most time in the center within 5 min of the start of the test, whereas the CSD group exhibited significant decreases for these two parameters. Notably, the CSDVD group exhibited slight increases in the number of entries into the center and the amount of time spent in the center.

The EPM is used to evaluate anxiety‐like behaviors, with a focus on the balance between risk‐taking and safety‐seeking behaviors. Figure 2G presents the results for the total distance traveled by the mice in this study. The CSD group exhibited a significantly shorter distance traveled than did the NS group, suggesting decreased locomotor activity. For this parameter, the CSDR and CSDVD groups did not exhibit significant differences relative to the CSD group. Figure 2H,I indicates a lack of any significant differences among the groups in terms of the percentage of entries into the closed arms and the percentage of entries into the open arms.

### 13.4 Effects of Vitamin D3 Supplementation and Sleep Recovery on Inflammation and Clock‐Related Gene Expression in Hypothalamus of Mice With Sleep Desynchrony

**FIGURE 3:**
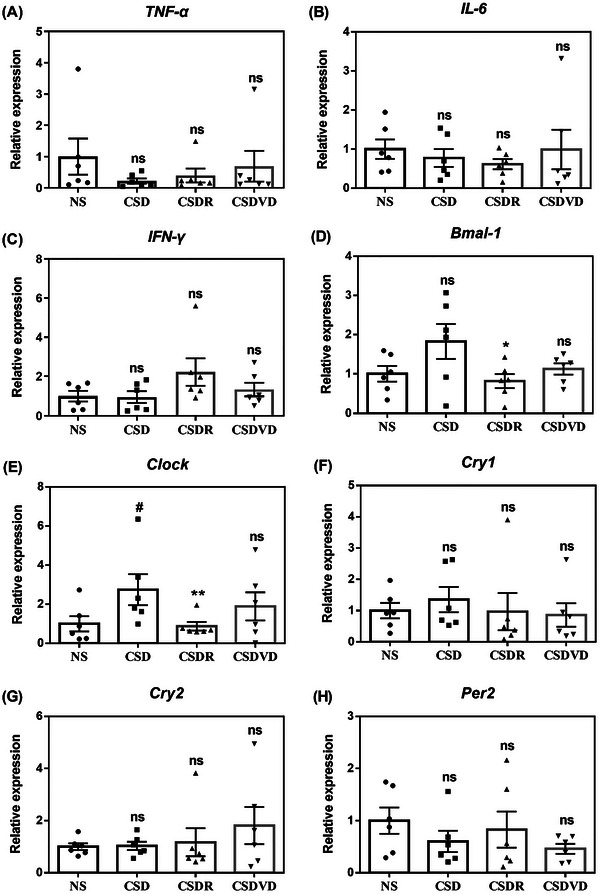
Effects of vitamin D3 treatment and sleep recovery on inflammatory genes and circadian genes of hypothalamus in mice with sleep desynchrony. (A) *TNF‐α*, (B) *IL‐6*, (C) *IFN‐*γ, (D) *Bmal‐1*, (E) *Clock*, (F) *Cry1*, (G) *Cry2*, and (H) *Per2*. NS: normal sleep group; CSD: chronic sleep deprivation group; CSDR: CSD followed by sleep recovery; CSDVD: CSD combined with vitamin D3 supplementation. Values are presented as means ± SEMs (*n* = 6). The unpaired one‐sided U test was performed for statistical analysis. #*p* < 0.05 compared with the NS group. *p < 0.05 and **p < 0.01 compared with the CSD group. ns indicates no statistical significance.

Figure 3 presents the results regarding the expression of inflammation and clock‐related genes in the hypothalamus. Regarding inflammatory gene expression, no significant differences in the expression levels of *TNF‐α*, *IL‐6*, and *IFN‐γ* were identified among the NS, CSD, CSDR, and CSDVD groups (Figure 3A–C). Analysis of clock‐related gene expression revealed that the CSD group exhibited higher expression levels of *Bmal‐1* and *clock* compared with the NS group; however, the increase in *Bmal‐1* expression did not reach statistical significance (Figure 3D,E), suggesting that sleep desynchrony upregulated these genes. Notably, both recovery and vitamin D3 treatment (CSDR and CSDVD groups) appeared to normalize *Bmal‐1* levels. There was no significant difference in *Cry1*, *Cry2*, and *Per2* expression among the groups (Figure 3F–H).

### 13.5 Effects of Vitamin D3 Supplementation and Sleep Recovery on White Blood Cell Counts in Mice With Sleep Desynchrony

**FIGURE 4:**
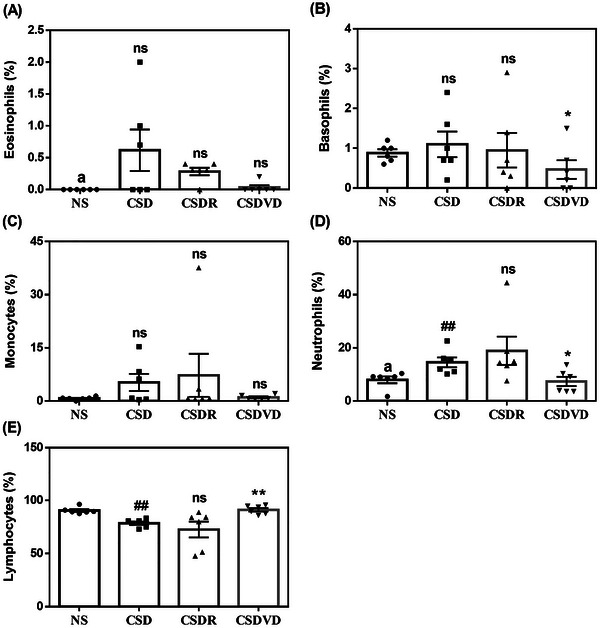
Effects of vitamin D3 treatment and sleep recovery on white blood cells in mice with sleep desynchrony. (A) Eosinophils, (B) basophils, (C) monocytes, (D) neutrophils, and (E) lymphocytes. NS: normal sleep group; CSD: chronic sleep deprivation group; CSDR: CSD followed by sleep recovery; CSDVD: CSD combined with vitamin D3 supplementation. Values are presented as means ± SEMs (*n* = 6). The unpaired one‐sided U test was performed for statistical analysis. ##*p* < 0.01 compared with the NS group. *p < 0.05 and **p < 0.01 compared with the CSD group. ns indicates no statistical significance.

As revealed in Figure 4A, the CSD group exhibited a slight increase in eosinophil percentage compared with the NS group. Both the CSDR and the CSDVD group exhibited slightly lower eosinophil percentages than did the CSD group. In addition, there were no significant differences in basophil percentages among the NS, CSD, and CSDR groups; however, the CSDVD group exhibited an obvious decrease relative to the CSD group (Figure 4B). As presented in Figure 4C, the CSD and CSDR groups exhibited slight increases in monocyte percentages relative to the NS group. By contrast, the CSDVD group exhibited a slightly lower percentage of monocytes than did the CSD group. As shown in Figure 4D, the CSD group significantly increased in neutrophil percentage compared with the NS group. However, the CSDVD group exhibited a significant decrease in neutrophil percentage relative to the CSD group. In addition, a decrease in lymphocyte percentage occurred in the CSD group relative to the NS group (Figure 4E). By contrast, the CSDVD group exhibited an increase in lymphocyte percentage compared with the CSD group. These findings reveal that sleep desynchrony increased and decreased the percentages of neutrophils and lymphocytes, respectively, indicating immune dysregulation. Vitamin D3 treatment significantly reduced neutrophil percentages but increased lymphocyte percentages, potentially restoring immune homeostasis.

### 13.6 Effects of Vitamin D3 Supplementation and Sleep Recovery on Immune Cell Modulation in Mice With Sleep Desynchrony

**FIGURE 5:**
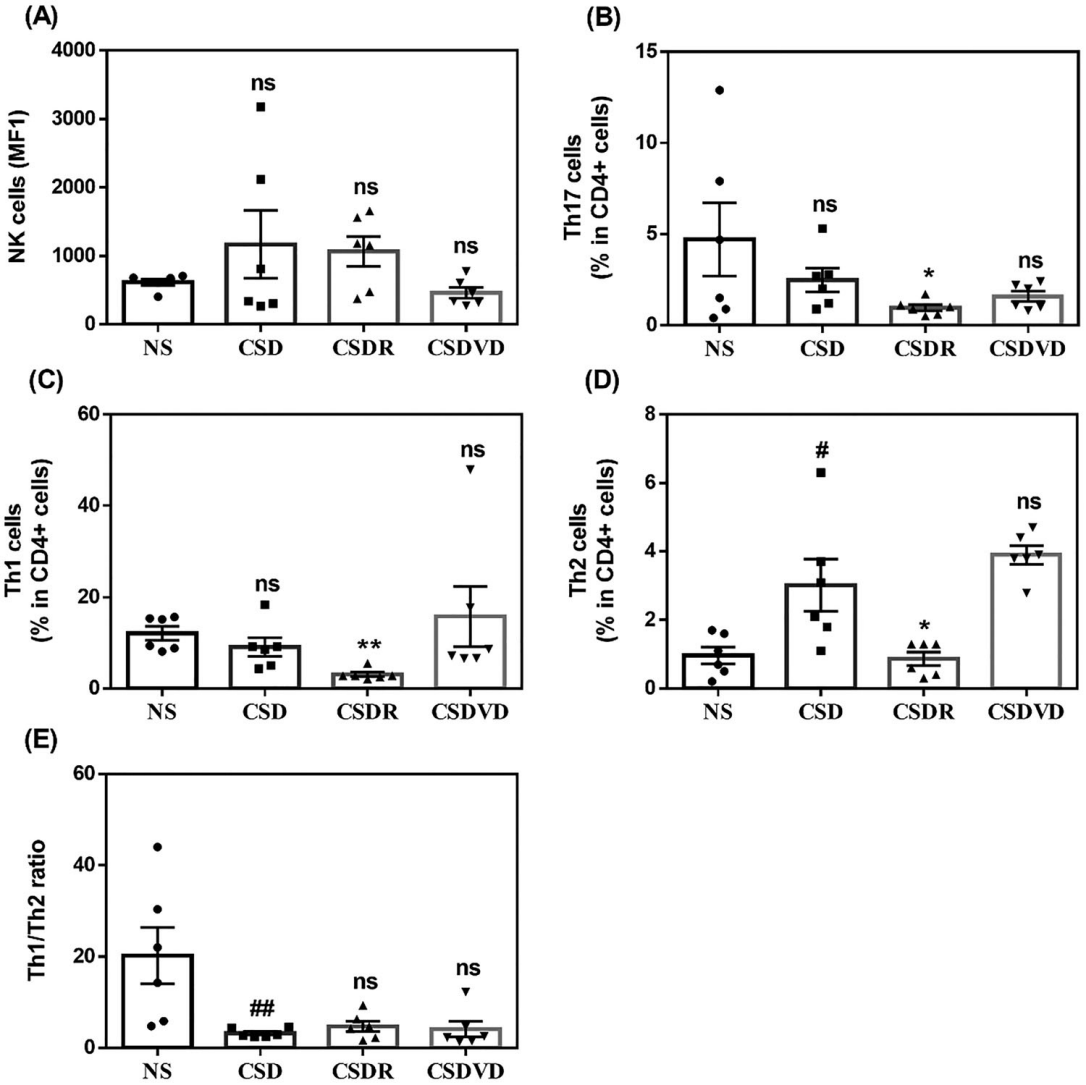
Effects of vitamin D3 treatment and sleep recovery on NK and T cells in mice with sleep desynchrony. (A) NK cells, (B) Th17 cells, (C) Th1 cells, (D) Th2 cells, and (E) Th1/Th2 ratio. NS: normal sleep group; CSD: chronic sleep deprivation group; CSDR: CSD followed by sleep recovery; CSDVD: CSD combined with vitamin D3 supplementation. Values are presented as means ± SEMs (*n* = 6). The unpaired one‐sided U test was performed for statistical analysis. #*p* < 0.05 and ##*p* < 0.01 compared with the NS group. *p < 0.05 and **p < 0.01 compared with the CSD group. ns indicates no statistical significance.

As indicated in Figure 5A, the CSD group exhibited a slight increase in NK cells relative to the NS group. By contrast, the CSDVD group exhibited a slight decrease in NK cells relative to the CSD group. Th17 cells, which are involved in inflammatory responses, were slightly decreased in the CSD group compared with the NS group (Figure 5B). Notably, sleep recovery significantly reduced Th17 cell levels. In addition, the CSDR group exhibited a significant reduction in Th1 cells compared with the CSD group (Figure 5C). The CSDVD group, which underwent treatment with vitamin D3, exhibited a slight increase in Th1 cells relative to the CSD group (Figure 5C). By contrast, the CSD group exhibited a significant increase in Th2 cells relative to the NS group (Figure 5D). Sleep recovery in the CSDR group reduced the Th2 cell percentage relative to the CSD group, whereas vitamin D3 treatment in the CSDVD group resulted in a slight increase in Th2 cells relative to the CSD group. Regarding the Th1/Th2 ratio (Figure 5E), the NS group exhibited the highest ratio, whereas the CSD group exhibited a significant decrease. These findings indicate that sleep recovery and vitamin D3 treatment did not affect the Th1/Th2 ratio compared with that in the CSD group.

### 13.7 Effects of Vitamin D3 Supplementation and Sleep Recovery on Compositions of Gut Microbiota in Mice With Sleep Desynchrony

**FIGURE 6:**
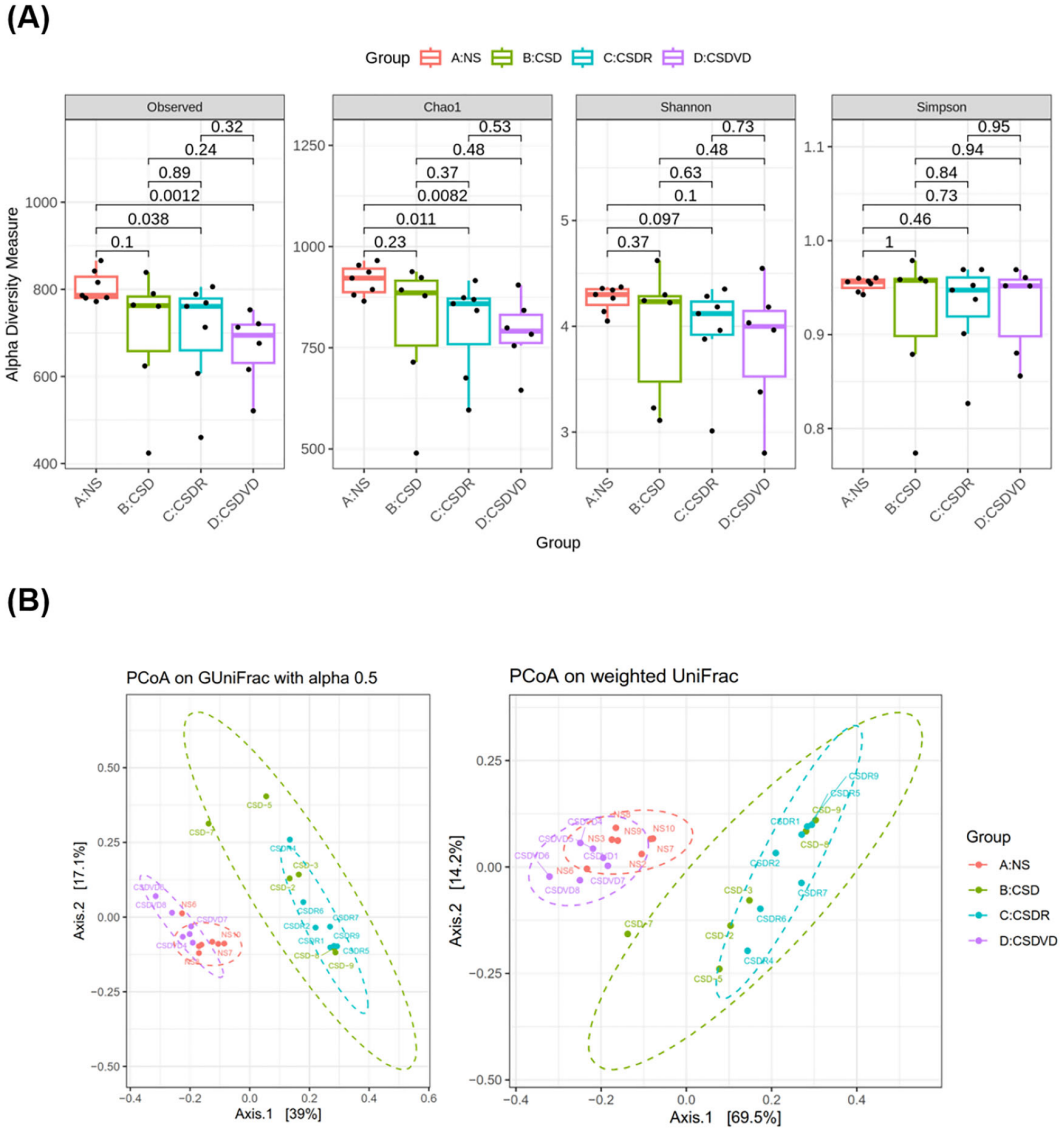
Effects of vitamin D3 treatment and sleep recovery on the diversity of gut microbiota in the feces of mice with sleep desynchrony. (A) Alpha diversity of observed species and Chao1, Shannon, and Simpson indices. (B) Results from beta diversity analysis based on GUniFrac with alpha 0.5 and weighted UniFrac principal coordinate analysis (PCoA) plots. Group differences were statistically assessed using PERMANOVA. NS: normal sleep group; CSD: chronic sleep deprivation group; CSDR: CSD followed by sleep recovery; CSDVD: CSD combined with vitamin D3 supplementation.

As presented in Figure 6A, no significant differences in the observed species or the Observed, Chao1, Shannon, and Simpson indices were identified among the NS, CSD, CSDR, and CSDVD groups. Regarding beta diversity, the differences in microbial composition between groups were visualized using PCoA based on GUniFrac with alpha 0.5 and weighted UniFrac (Figure 6B). The PCoA plots reveal a distinct clustering of gut microbiota composition between the NS, CSD, CSDR, and CSDVD groups, with the CSD group forming a cluster clearly separated from the NS group, indicating a shift in microbial community structure due to sleep desynchrony. The CSDVD group, which underwent vitamin D3 treatment, exhibited a microbial composition closer to that of the NS group, suggesting that vitamin D3 mitigates the dysbiotic effects of circadian disruption. Notably, the CSDR group cluster remained close to the CSD group cluster, indicating that recovery of gut microbiota through sleep recovery alone was minimal.

**FIGURE 7:**
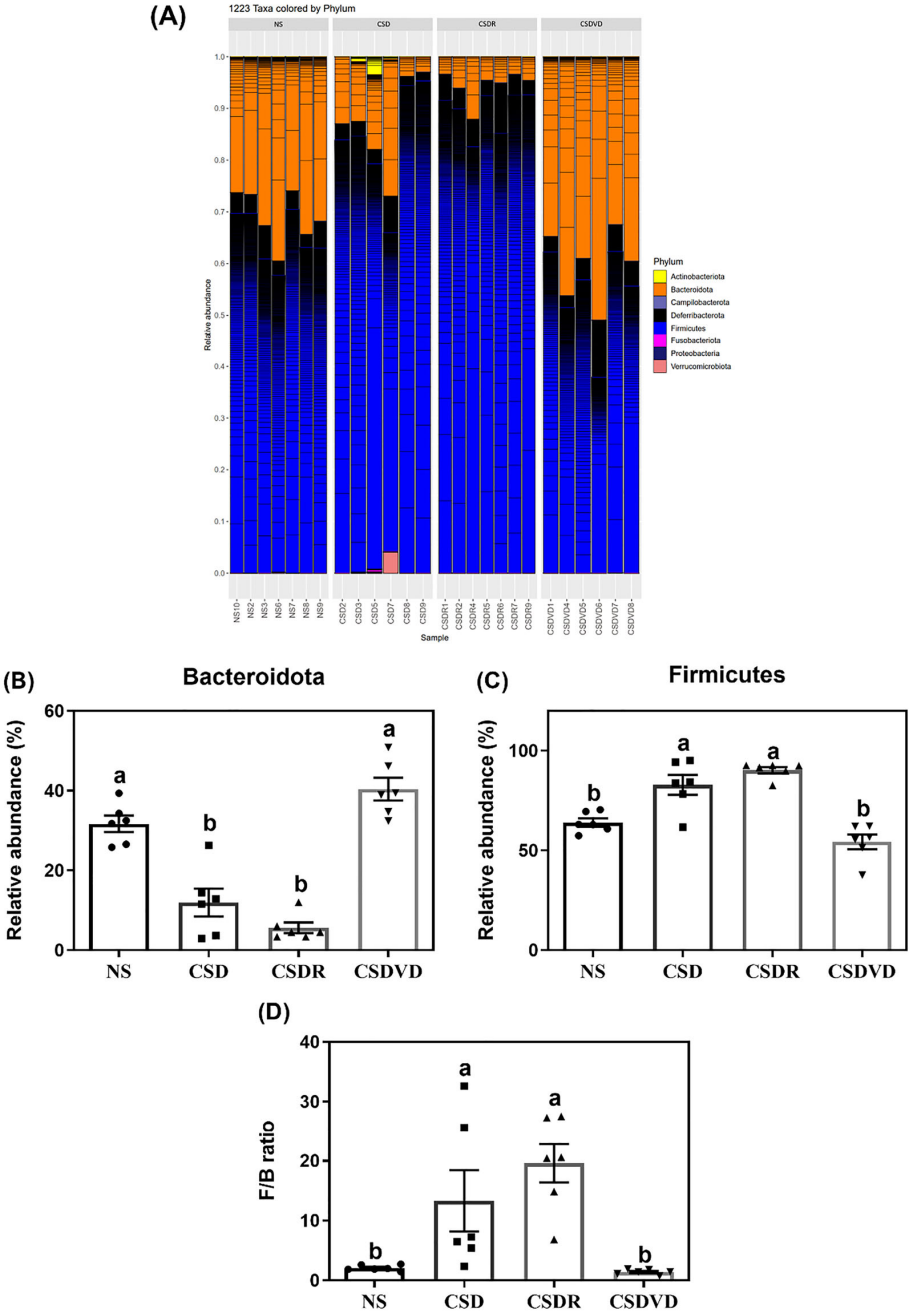
Effects of vitamin D3 treatment and sleep recovery on the diversity of gut microbiota in the feces of mice with sleep desynchrony. Relative abundances of microbiota species: (A) phylum level, (B) Bacteroidota, (C) Firmicutes, and (D) F/B ratio. NS: normal sleep group; CSD: chronic sleep deprivation group; CSDR: CSD followed by sleep recovery; CSDVD: CSD combined with vitamin D3 supplementation. Values are presented as means ± SEMs (*n* = 6). The unpaired one‐sided U test was performed for statistical analysis. #*p* < 0.05 and ##*p* < 0.01 compared with the NS group. **p < 0.01 compared with the CSD group. ns indicates no statistical significance.

The microbiota composition at the phylum level was significantly different among the NS, CSD, CSDR, and CSDVD groups (Figure 7A). As illustrated in Figure 7B, the CSD group exhibited a significant reduction in Bacteroidota compared with the NS group. Notably, sleep recovery alone did not significantly restore Bacteroidota. Nevertheless, the CSDVD group exhibited a significant increase in Bacteroidota relative to the CSD group. As indicated in Figure 7C, the CSD group exhibited a significant increase in Firmicutes relative to the NS group. Similar to the results for Bacteroidota, those for Firmicutes did not indicate a significant change in the CSDR group. However, the CSDVD group exhibited a significant reduction in Firmicutes relative to the CSD group. As shown in Figure 7D, the Firmicutes/Bacteroidota (F/B) ratio was notably elevated in the CSD group compared with the NS group, reflecting the dysbiosis induced by sleep desynchrony. These results indicate that sleep recovery alone did not significantly alter the F/B ratio, whereas vitamin D3 treatment significantly reduced the F/B ratio, bringing it closer to NS group levels.

**FIGURE 8:**
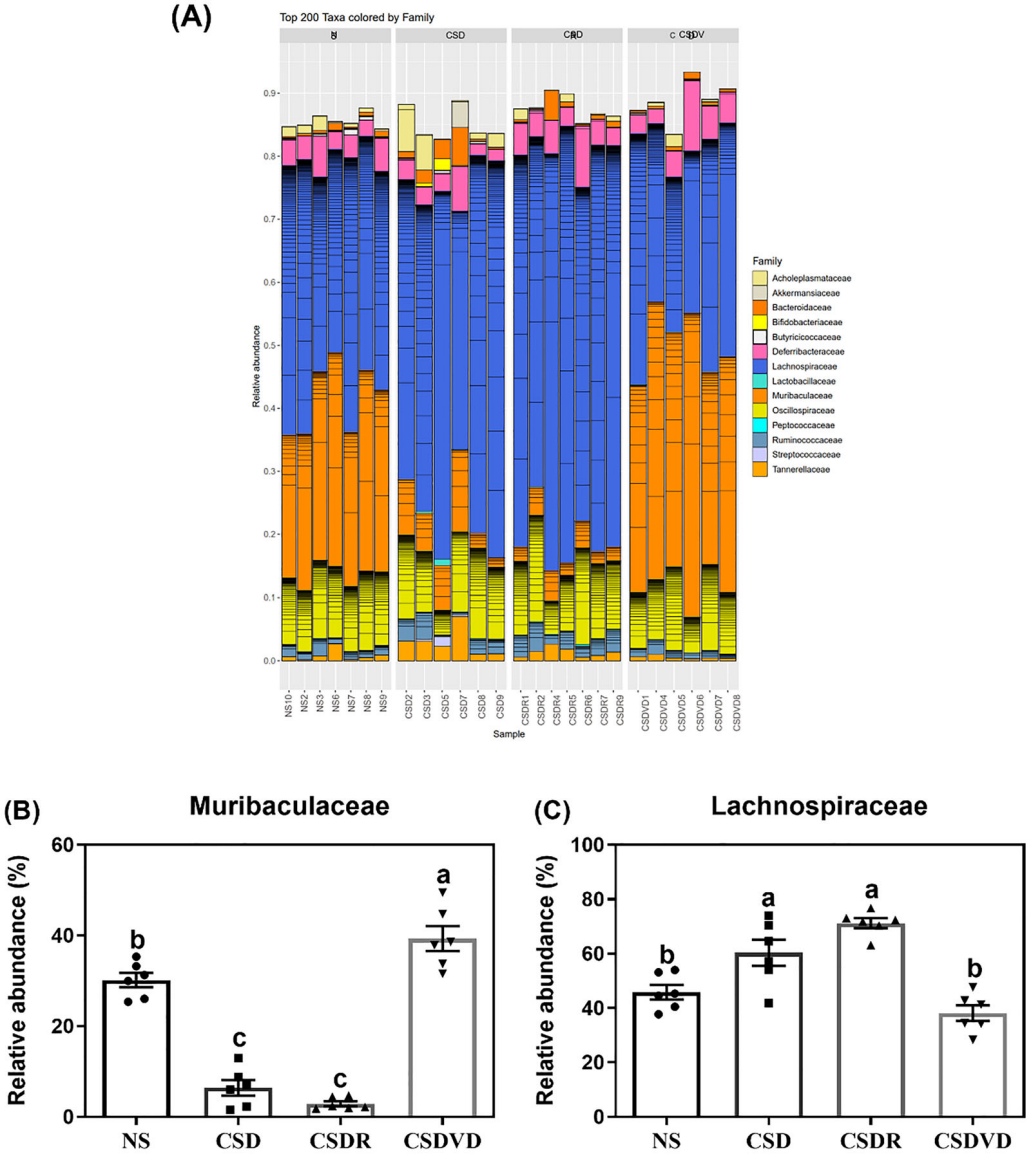
Effects of vitamin D3 treatment and sleep recovery on the diversity of gut microbiota in the feces of mice with sleep desynchrony. Relative abundances of microbiota species: (A) family level, (B) Lachnospiraceae, and (C) Muribaculaceae. NS: normal sleep group; CSD: chronic sleep deprivation group; CSDR: CSD followed by sleep recovery; CSDVD: CSD combined with vitamin D3 supplementation. Values are presented as means ± SEMs (*n* = 6). The unpaired one‐sided U test was performed for statistical analysis. #*p* < 0.05 and ##*p* < 0.01 compared with the NS group. **p < 0.01 compared with the CSD group. ns indicates no statistical significance.

Figure 8A presents the family‐level results, revealing that compared with the NS group, the CSD group exhibited an increased percentage of Lachnospiraceae (Figure 8B) and a decreased percentage of Muribaculaceae (Figure 8C). The percentages of Lachnospiraceae and Muribaculaceae in the CSDR group remained largely unchanged. However, vitamin D3 treatment significantly reduced and increased the levels of Lachnospiraceae and Muribaculaceae, respectively, aligning them more closely with the NS group levels.

**FIGURE 9:**
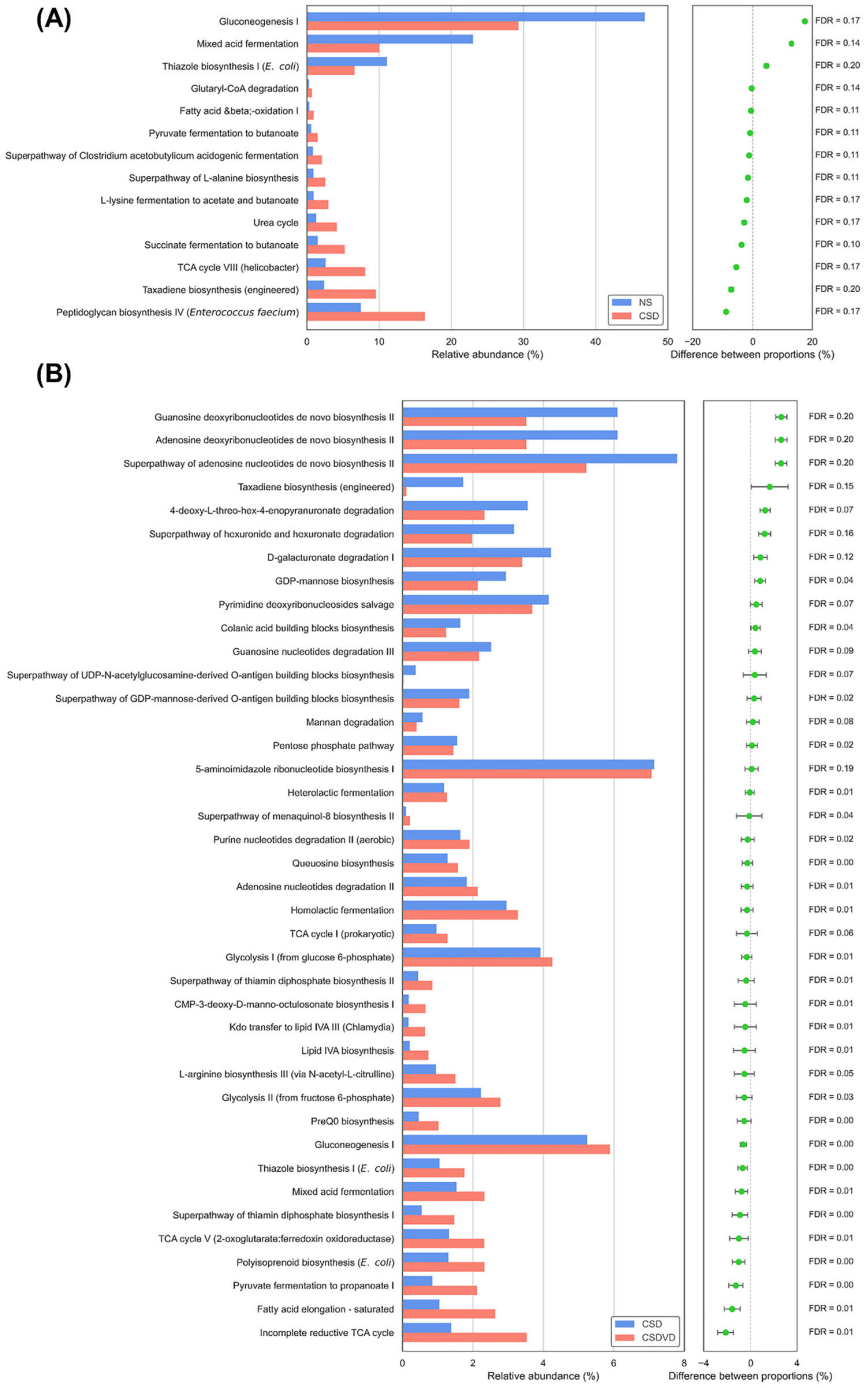
Effects of vitamin D3 treatment on predicted microbial functional pathways using PICRUSt2 based on 16S rRNA sequencing data. (A) Differentially enriched MetaCyc pathways in CSD mice compared with NS mice, reflecting the impact of sleep desynchrony. (B) Differentially enriched MetaCyc pathways in CSDVD mice compared with CSD mice, reflecting the effects of vitamin D3 supplementation. Pathways were identified based on effect size and statistical significance determined by Welch's *t*‐test with Benjamini–Hochberg false discovery rate (FDR) correction. Bar length represents relative abundance. NS: normal sleep group; CSD: chronic sleep deprivation group; CSDR: CSD followed by sleep recovery; CSDVD: CSD combined with vitamin D3 supplementation.

As shown in Figure 9A, compared with the normal sleep group (NS), the sleep deprivation group (CSD) exhibited a notable reduction in microbial functional pathways related to energy metabolism, including Gluconeogenesis I, Mixed acid fermentation, and Thiazole biosynthesis I (*E. coli*). In contrast, Peptidoglycan biosynthesis IV (*Enterococcus faecium*) was elevated in the CSD group, potentially reflecting a bloom of specific taxa and increased bacterial immunogenicity. Although several of these differences demonstrated borderline statistical significance (FDR range: 0.10–0.20), they may still represent biologically meaningful disruptions in microbial metabolic capacity induced by chronic sleep desynchrony. Following vitamin D3 supplementation, as presented in Figure 9B, many of these pathway alterations were partially restored. The CSDVD group showed increased abundance in pathways involved in energy metabolism and amino acid biosynthesis, including Glycolysis I (from glucose 6‐phosphate), Glycolysis II (from fructose 6‐phosphate), and L‐arginine biosynthesis III. Conversely, the abundance of Mixed acid fermentation and Thiazole biosynthesis (*E. coli*) was increased compared to the CSD group, suggesting a shift away from pro‐inflammatory microbial activity. While not all differences met strict significance thresholds, the observed trends support the notion that vitamin D3 modulates microbiota‐derived functional pathways relevant to metabolic and neuroimmune homeostasis under circadian stress.

### 13.8 Effects of Vitamin D3 Supplementation and Sleep Recovery on Expression of Tight Junction Proteins, Endotoxin, and Fecal SCFAs in Mice With Sleep Desynchrony

**FIGURE 10:**
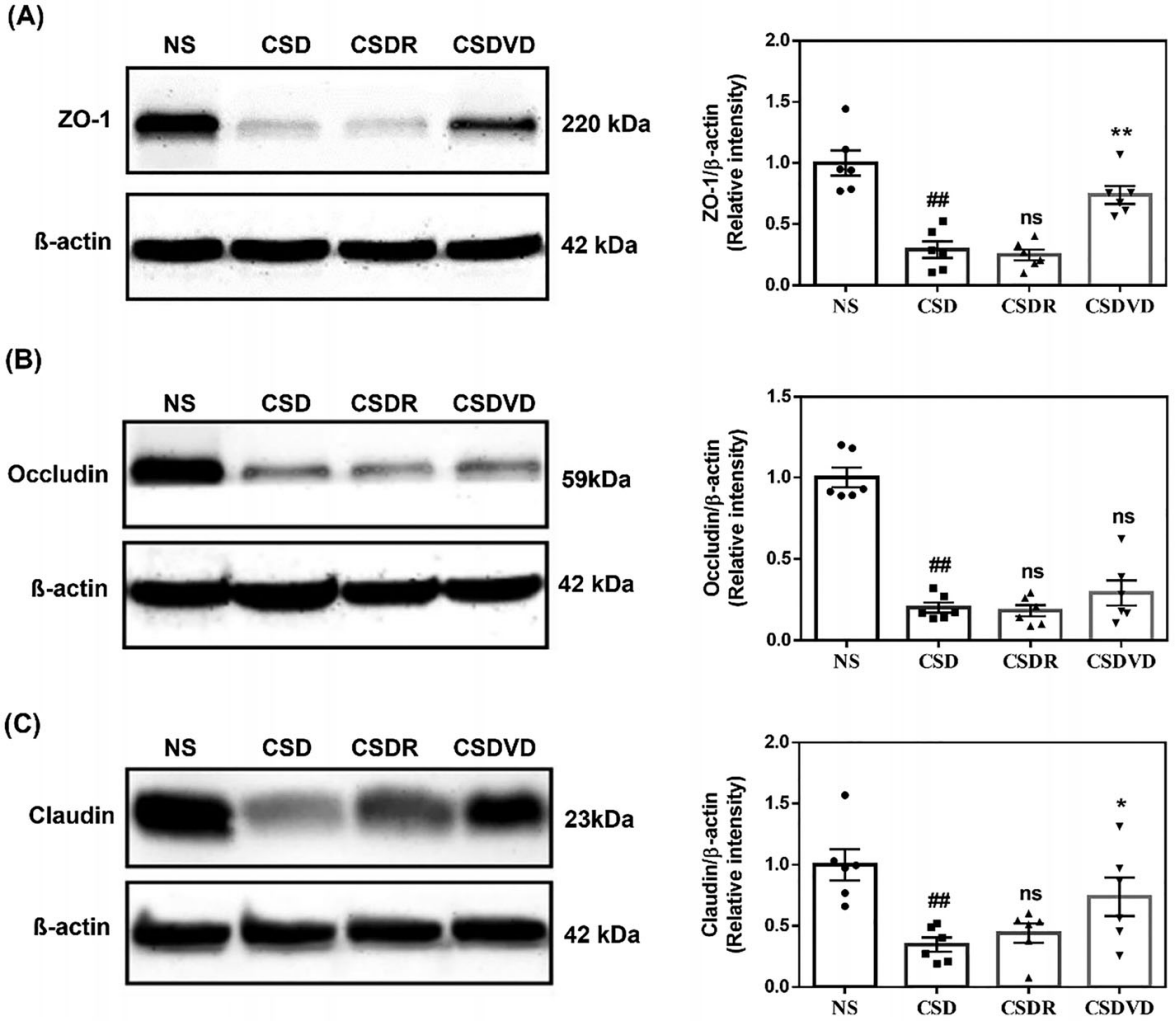
Effects of vitamin D3 treatment and sleep recovery on the expression of tight junction proteins in the colon of mice with sleep desynchrony. Expression levels: (A) ZO‐1, (B) occludin, and (C) claudin. NS: normal sleep group; CSD: chronic sleep deprivation group; CSDR: CSD followed by sleep recovery; CSDVD: CSD combined with vitamin D3 supplementation. Values are presented as means ± SEMs (*n* = 6). The unpaired one‐sided U test was performed for statistical analysis. ##*p* < 0.01 compared with the NS group. *p < 0.05 and **p < 0.01 compared with the CSD group. ns indicates no statistical significance.

To evaluate the effect of sleep desynchrony on the integrity of tight junction proteins in the proximal colon of mice, the present study examined the expression levels of ZO‐1 (Figure 10A), occludin (Figure 10B), and claudin‐1 (Figure 10C) proteins. In the CSD group, the expression of ZO‐1 was significantly reduced compared with the NS group. However, its ZO‐1 level was significantly restored to near‐normal levels after vitamin D3 treatment but not in the CSDR group. The CSD group exhibited significantly reduced occludin levels compared with the NS group. However, no significant differences in occludin levels were identified among the CSD, CSDR, and CSDVD groups. As indicated in Figure 10C, the claudin levels were also significantly reduced in the CSD group compared with the NS group. Notably, vitamin D3 treatment significantly increased claudin levels in the CSDVD group but not in the CSDR group. This finding suggests that sleep desynchrony led to a significant reduction in the expression of tight junction proteins (ZO‐1, occludin, and claudin) in the proximal colon. Vitamin D3 treatment effectively restored ZO‐1 and claudin levels, whereas sleep recovery alone did not result in substantial improvements. These findings suggest that vitamin D3 supplementation plays a protective role in maintaining intestinal barrier integrity under sleep desynchrony.

**FIGURE 11:**
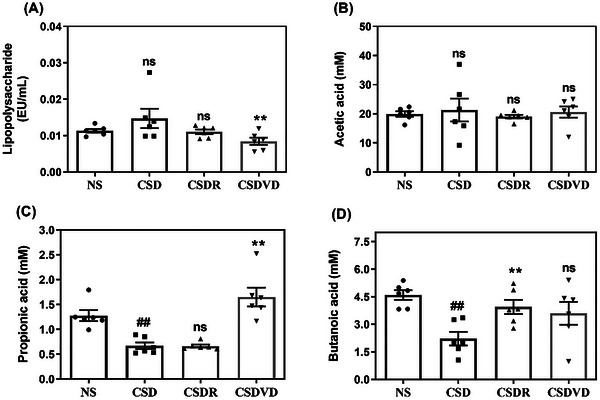
Effects of vitamin D3 treatment and sleep recovery on levels of serum lipopolysaccharide and short chain fatty acids in mice with sleep desynchrony. (A) Lipopolysaccharide, (B) acetic acid, (C) propionic acid, and (D) butanoic acid. NS: normal sleep group; CSD: chronic sleep deprivation group; CSDR: CSD followed by sleep recovery; CSDVD: CSD combined with vitamin D3 supplementation. Values are presented as means ± SEMs (*n* = 6). The unpaired one‐sided U test was performed for statistical analysis. ##*p* < 0.01 compared with the NS group. **p < 0.01 compared with the CSD group. ns indicates no statistical significance.

Blood LPS levels in the NS, CSD, CSDR, and CSDVD groups were measured to determine whether sleep desynchrony led to damage in tight junction proteins and a subsequent increase in LPS levels (Figure 11A). The results showed that the blood LPS level of the CSD group was slightly elevated compared with the NS group. At the same time, its blood LPS level was significantly restored to near‐normal level after vitamin D3 treatment but not in the CSDR group.

SCFAs are key metabolites produced by gut microbiota, and they play crucial roles in maintaining intestinal barrier integrity and immune regulation. As revealed in Figure 11B, no significant differences were noted in the acetic acid levels among the NS, CSD, CSDR, and CSDVD groups. In addition, the CSD group exhibited a significant reduction in propionic acid levels relative to the NS group (Figure 11C). In the CSDVD group, vitamin D3 treatment restored propionic acid levels to near‐normal values. As shown in Figure 11D, the CSD group exhibited a significant reduction in butanoic acid levels relative to the NS group. The CSDR group exhibited a significant increase in butanoic acid levels compared with the CSD group, while the CSDVD group showed a modest increase.

## 14 Discussion

The findings of the present study reveal that 28 days of sleep desynchrony did not significantly alter the body weight, food intake, or water consumption in the mice. The mice in all groups (NS, CSD, CSDR, and CSDVD) exhibited normal and steady increases in body weight over the course of the experiment, and no significant differences between initial and final body weight were identified among the groups. Similarly, no significant differences in food and water intake were detected, suggesting that prolonged sleep disruption did not substantially influence the fundamental metabolic processes in these animals. Despite experiencing extended periods of circadian misalignment, the mice appeared to maintain metabolic homeostasis. This stability may be attributable to the capacity of the mice to adaptively regulate energy expenditure in response to sustained stress, thereby ensuring the preservation of vital physiological functions; the mice may have employed intrinsic regulatory mechanisms to offset energy deficits induced by CSD.

The secretion of corticosterone, a key stress hormone, is regulated by the HPA axis, and its function can be evaluated using the dexamethasone suppression test. In the NS group, the administration of dexamethasone led to a significant decrease in corticosterone levels, indicating a normal HPA axis suppression response. However, in the CSD group, dexamethasone failed to effectively suppress corticosterone secretion, suggesting HPA axis dysfunction. This could have resulted from a weakened negative feedback mechanism, reflecting desensitization or tolerance of the HPA axis after chronic sleep desynchrony. Notably, in the mice treated with vitamin D3, dexamethasone significantly suppressed corticosterone secretion, indicating that vitamin D3 supplementation helped to ameliorate HPA axis dysfunction induced by sleep desynchrony. The beneficial effects of vitamin D3 may stem from its anti‐inflammatory and antioxidant properties (Silva and Lazaretti‐Castro 2022). Studies have reported that emotional disorders such as anxiety and depression are often associated with a reduction in antioxidant enzyme activity in the HPA axis (Rasmus and Kozłowska 2023). Therefore, as an antioxidant, vitamin D3 may enhance antioxidant enzyme activity, thereby restoring the HPA axis's sensitivity to stress. One study also suggests that vitamin D helps to regulate the stress axis (Rolf et al. 2018). In addition, vitamin D3 was revealed to suppress glucocorticoid‐induced transcription and cytotoxicity in hippocampal cell cultures (Eyles et al. 2005). Studies focused on the human central nervous system have reported that the highest expression of vitamin D receptors and activating enzymes occurred in the hypothalamus, particularly in the paraventricular nucleus (Eyles et al. 2005; Smolders et al. 2013). Smolders et al. (2013) indicated that the corticotrophin‐releasing hormone‐positive neurons in the paraventricular nucleus, which are part of the stress axis, also express vitamin D 24‐hydroxylase (Smolders et al. 2013), suggesting that these neurons are responsive to vitamin D. Our finding is consistent with those of previous studies on the role of vitamin D3 in maintaining stress homeostasis and protecting against stress‐related damage. Moreover, in the current study, after 28 days of sleep desynchrony, no significant differences in baseline corticosterone levels were identified among the NS, CSD, and CSDVD groups. This suggests that sleep desynchrony impairs HPA axis regulation but does not lead to significant changes in baseline corticosterone levels in the short term. These results indicate that vitamin D3 treatment can effectively improve HPA axis dysfunction caused by sleep desynchrony and restore normal stress response regulation.

Although vitamin D3 supplementation exerted beneficial effects on behavior, immune responses, and gut barrier integrity, its impact on central stress regulation—particularly hypothalamic cytokine expression and HPA axis normalization—remained limited. This partial restoration may reflect the complexity of neuroendocrine feedback circuits and the need for additional interventions to fully restore central sensitivity. To enhance HPA axis resilience, several adjunctive strategies have shown promise in modulating stress responsivity via complementary mechanisms. Adaptogens—natural compounds such as Rhodiola and Schisandra—have been reported to stabilize HPA axis function by attenuating excessive glucocorticoid release and normalizing stress‐induced neuroendocrine signals, including heat shock proteins and FOXO transcription factors (Tóth‐Mészáros et al. 2023). Melatonin, a well‐established chronobiotic, not only entrains circadian rhythms but also directly inhibits CRH and ACTH secretion, thereby dampening corticosterone elevation under acute and chronic stress (Konakchieva et al. 1997). Additionally, psychobiotics such as *Lactiplantibacillus plantarum* PS128 can modulate the gut–brain axis via vagal afferents and immune signaling, leading to reduced peripheral cortisol levels and improved stress resilience (Wu et al. 2021b). Omega‐3 polyunsaturated fatty acids (PUFAs), notably EPA and DHA, have been shown to attenuate neuroinflammation, enhance glucocorticoid receptor sensitivity, and normalize HPA axis function through hippocampal modulation (Zhou et al. 2022a). These interventions may synergize with vitamin D3 by targeting distinct levels of HPA regulation—omega‐3s reinforcing receptor‐level feedback sensitivity, psychobiotics modulating upstream stress perception, and adaptogens or melatonin stabilizing central output. Therefore, future studies should consider combinatorial approaches incorporating vitamin D3 with these agents to achieve more comprehensive restoration of neuroendocrine homeostasis under chronic circadian disruption.

In the present study, behavioral tests were used to evaluate the effects of sleep desynchrony on cognition, locomotor activity, and anxiety‐like behaviors in mice, with a focus on the effects of vitamin D3 treatment. In the present study's experiment, the NS and CSDVD mice constructed higher‐quality nests with more complete structures, suggesting improved overall health in these groups. By contrast, the CSD group exhibited a slight decline in nest‐building ability, suggesting that sleep desynchrony negatively affects motivation and physical condition. By contrast, the CSDVD group exhibited improvements in nest quality, suggesting that vitamin D3 supplementation can restore cognitive function and motivation. In addition, the OFT results indicated that the NS group exhibited greater locomotor activity, as evidenced by a wider range of movement within the test arena. By contrast, the CSD group exhibited a significant reduction in movement range, suggesting that sleep desynchrony impaired the mice's locomotor capacity and exploratory behavior. The CSDR and CSDVD groups exhibited partial recovery of activity levels. In particular, the CSDVD group exhibited a significant increase in distance traveled compared with the CSD group. This suggests that vitamin D3 plays a role in improving locomotor function and reducing anxiety‐like behaviors. In the EPM test, the CSD group exhibited a significant reduction in total distance traveled, which is consistent with the observed reduction in locomotor activity. By contrast, the CSDR and CSDVD groups did not exhibit significant differences in total distance traveled relative to the CSD group.

Although the EPM, OFT, and NBTs are commonly used to assess anxiety‐like behaviors in mice, they each reflect distinct behavioral domains. The OFT and NBTs are more sensitive to changes in general activity, motivation, and mild anxiety, whereas the EPM focuses on approach–avoidance conflict and risk assessment under high‐stress conditions. As noted by Figueiredo Cerqueira et al. (2023), the OFT captures exploratory behavior in novel environments, while the EPM reflects behavioral inhibition in threatening contexts. However, Shoji and Miyakawa (2021) reported that EPM results are more susceptible to environmental factors and prior testing experience, potentially limiting their consistency. Therefore, the absence of significant differences in the EPM in this study—despite anxiolytic‐like trends observed in the OFT and NBTs—may be due to the EPM's distinct sensitivity to risk‐related behaviors. This highlights the importance of using multiple behavioral assays to capture various aspects of anxiety.

A study reported that insufficient vitamin D intake is linked to various mental disorders, including anxiety (McCann and Ames 2008). Another reported that moderate‐dose vitamin D supplementation (≥800 IU daily) had beneficial effects in treating depression, particularly when serum vitamin D levels were lower than the normal range (Spedding 2014). Additionally, vitamin D supplementation over a period of 2 years was associated with improved sleep in 1500 patients experiencing neurological conditions (Gominak and Stumpf 2012). A double‐blind clinical trial demonstrated that vitamin D3 supplementation significantly improved anxiety, depression, and physical health in university students but had no significant effect on sleep quality (Karami‐Mohajeri et al. 2021). By contrast, another study did not identify any significant differences between the vitamin D3 and placebo groups in terms of depression incidence, recurrence, or changes in mood scores, suggesting that vitamin D3 supplementation did not help prevent depression (Okereke et al. 2020). In the present study, the mice that received vitamin D3 supplementation built higher‐quality nests and exhibited improved locomotor activity relative to the other groups, suggesting that vitamin D3 promoted cognitive recovery and motor function. Notably, the potential of vitamin D3 to improve anxiety and depression has been supported by other studies, such as those reporting that moderate doses of vitamin D supplementation helped to treat depression, particularly when vitamin D levels were lower than normal. However, several long‐term studies have indicated that vitamin D3 has limited preventive effects on depression and does not significantly improve sleep quality. Emerging evidence suggests that both higher dosages and longer durations of vitamin D intervention may be required to elicit more robust neurobehavioral and immunomodulatory outcomes. For instance, Gao et al. (2018) reported that high‐dose vitamin D supplementation led to significant improvements in depressive symptoms in individuals with major depressive disorder. A recent preclinical study showed that extending vitamin D3 treatment duration in rats significantly improved neuroinflammation and behavioral deficits induced by chronic stress (Arabshahi et al. 2024). Similarly, a clinical study in Iranian students found greater improvements in psychological well‐being after extended administration of higher‐dose vitamin D3 (Mansouri et al. 2024). Therefore, although the results of the present study suggest that vitamin D3 supplementation contributes to the partial recovery of locomotor function, its role in regulating anxiety may be limited in the short term. Future studies with varied doses and longer intervention duration should be conducted to enable more comprehensive assessment of such effects.

Sleep desynchrony led to an increase in neutrophils and a decrease in lymphocytes, indicating a shift in the immune system toward a more pro‐inflammatory state. Vitamin D3 treatment helped to counteract this by reducing neutrophils and increasing lymphocytes, thereby contributing to the re‐establishment of immune homeostasis. A study reported an inverse relationship between 25‐hydroxyvitamin D levels and inflammation, as indicated by a negative correlation between vitamin D levels and the neutrophil‐to‐lymphocyte ratio (Akbas et al. 2016). Collectively, these findings highlight the potential of vitamin D3 in modulating immune responses and reducing inflammation. Regarding immune cell modulation, CSD slightly increased the number of NK cells and altered the proportion of T helper cells, both of which play crucial roles in regulating immune responses, and sleep recovery reduced Th17 cells. Notably, although Th1 cells significantly decreased after sleep recovery, vitamin D3 treatment appeared to slightly elevate the levels of these cells, suggesting it plays a role in balancing Th1 and Th2 responses. However, it did not fully restore the Th1/Th2 ratio to normal levels. These findings suggest that sleep desynchrony significantly affected immune function, shifting immune cell populations toward a more inflammatory state. However, the effects of vitamin D3 on specific immune cells, such as Th1, Th2, and NK cells, appeared to be limited.

In this study, sleep desynchrony did not significantly alter the expression of inflammation‐related genes in the hypothalamus, as evidenced by the absence of significant differences in the expression levels of inflammatory factors, such as *TNF‐α*, *IL‐6*, and *IFN‐γ*, among the NS, CSD, CSDR, and CSDVD groups. This suggests that short‐term sleep desynchrony did not induce a pronounced systemic inflammatory response. However, the CSD group exhibited a significant increase in the expression of *clock* genes, indicating that sleep desynchrony affected the expression of circadian gene. This finding further highlights the close interaction between sleep and circadian rhythms. The upregulation of these genes may be related to an imbalance in the internal time regulation mechanism caused by sleep disruption. Notably, both sleep recovery and vitamin D3 treatment restored *Bmal‐1* expression to near‐normal levels, suggesting that these interventions had a positive effect on the normal functioning of circadian genes. Therefore, although sleep desynchrony did not significantly affect the expression of inflammatory genes in the hypothalamus, it significantly affected the expression patterns of circadian genes.

This study analyzed the effect of sleep desynchrony on gut microbiota composition and the potential role of vitamin D3 in regulating gut microbiota imbalance. Although no significant differences in alpha diversity indices (e.g., observed species and Chao1, Shannon, and Simpson indices) were identified among the NS, CSD, CSDR, and CSDVD groups, beta diversity analysis (based on GUniFrac and weighted UniFrac PCoA plots) revealed significant differences in gut microbiota composition between the CSD and NS groups. This indicates that sleep desynchrony altered the structure of the gut microbiome. Notably, the gut microbiota composition in the CSDVD group, which underwent vitamin D3 treatment, shifted closer to that of the NS group, suggesting that vitamin D3 treatment helps mitigate dysbiosis induced by circadian rhythm disruption. By contrast, the microbiota composition in the CSDR group, which underwent sleep recovery alone, remained similar to that of the CSD group, indicating that sleep recovery alone had limited effects on restoring gut microbiota. At the phylum level, the CSD group exhibited a significant reduction in Bacteroidota and an increase in Firmicutes, indicating gut dysbiosis due to sleep desynchrony. Vitamin D3 treatment significantly increased Bacteroidota and decreased the Firmicutes percentage in gut microbiota. Also, this study observed a notable increase in the F/B ratio in the CSD group that reflected a severe microbiome imbalance. Vitamin D3 treatment significantly reduced this ratio, bringing it closer to the levels observed in the NS group. Ciubotaru et al. (2015) suggested that vitamin D3 supplementation alters the microbiota composition by reducing the abundance of the phylum Firmicutes. In contrast, Mandal et al. (2016) reported that vitamin D treatment can increase the Proteobacteria/Firmicutes ratio. However, both studies reported an increase in the population of Bacteroidetes.

At the family level, the increase in Lachnospiraceae and the decrease in Muribaculaceae in the CSD group indicated gut microbiota dysregulation, but vitamin D3 treatment effectively reversed these changes, confirming its role in re‐establishing gut microbial balance. One study (Ko et al. 2019) reported an increase in the relative abundance of Lachnospiraceae when the severity of sleep apnea–hypopnea syndrome increased. In another study, the relative abundance of Lachnospiraceae was, on average, negatively correlated with sleep efficiency and total sleep time (Smith et al. 2019). One study suggested that vitamin D is associated with specific genera within the Lachnospiraceae family, including *Blautia*, *Roseburia*, *Dorea*, and *Coprococcus* (Tangestani et al. 2021). Muribaculaceae, which belongs to the phylum Bacteroidetes, is a beneficial bacterium found within the intestinal microbiota of mice. Our previous study indicated that the relative abundance of Muribaculaceae was lower in mice subjected to acute sleep deprivation (Yang et al. 2023). Several studies have demonstrated that this family contributes to the extension of lifespan in mice (Lagkouvardos et al. 2019; Smith et al. 2021). Studies involving ruminants have reported that the relative abundance of Muribaculaceae correlates significantly with circadian oscillations in the rumen of dairy cows because of the influence of melatonin, and its higher abundance has been linked to potential benefits in treating subclinical mastitis in these animals (Ouyang et al. 2021; Wang et al. 2021). However, another study reported a reduction in Muribaculaceae in hemodialysis patients with sarcopenia (Zhou et al. 2022b). Yet another study reported that short‐term calcitriol treatment did not reduce intestinal bacterial overgrowth but induced significant changes in bacterial diversity and promoted the enrichment of Muribaculaceae, Bacteroidales, Allobaculum, Anaerovorax, and Ruminococcaceae (Lee et al. 2021).

Notably, the PICRUSt‐predicted functional alterations are consistent with recent studies showing that sleep deprivation disrupts gut microbial composition, reduces beneficial taxa, and promotes the expansion of opportunistic bacteria, which are associated with intestinal inflammation and impaired barrier function (Wang et al. 2022; Liu et al. 2025). Decreased gluconeogenesis and fermentation pathways may reflect impaired microbial SCFAs output, disrupting epithelial energy supply and immune balance (Tremaroli and Bäckhed 2012). Furthermore, recent evidence indicates that brain peptidoglycan (PG) levels are dynamically regulated by sleep and wake states as well as circadian rhythms (English and Krueger 2025). For instance, PG concentrations decrease in regulatory regions such as the hypothalamus and brainstem during acute sleep deprivation, but increase in cortical areas, suggesting region‐specific shifts in PG localization. Sleep deprivation also alters the expression of PG recognition proteins, such as Pglyrp1, implying heightened host immune surveillance under disrupted sleep conditions (Rehman et al. 2001). These changes may trigger neuroimmune cascades that sustain low‐grade inflammation. The observed enrichment in bacterial polysaccharide biosynthesis and Mixed acid fermentation pathways may contribute to increased microbial immunogenicity and enhanced inflammatory signaling, reflecting a greater antigenic potential. These bacterial structural components may activate host pattern recognition receptors and initiate mucosal immune responses, contributing to sustained low‐grade inflammation. In particular, metabolites derived from bacterial polysaccharide biosynthesis, such as LPS and capsular polysaccharides, have been shown to cross a compromised gut–brain barrier and stimulate microglial activation, contributing to neuroinflammation (Farmen et al. 2021). Similarly, end products of Mixed acid fermentation, including acetate, succinate, and propionate, can modulate glial cell activity and cytokine release in the central nervous system via G‐protein‐coupled receptors or histone deacetylase inhibition (Blad et al. 2012). Collectively, these findings suggest that the activation of pro‐inflammatory microbial pathways under conditions of sleep desynchrony and vitamin D3 supplementation may reflect a neuroimmune response linking microbial metabolism to host inflammatory tone and sleep regulation.

The observed enrichment in microbial pathways related to amino acid biosynthesis and glycolysis following vitamin D3 supplementation may underlie the behavioral improvements seen in the CSDVD group. Notably, the upregulation of L‐arginine biosynthesis III in the CSDVD group suggests enhanced microbial contribution to systemic arginine availability. L‐arginine is a semi‐essential amino acid critical not only for protein synthesis but also as a precursor for nitric oxide (NO), a mediator of neuronal communication, vascular tone, and immune regulation (Wu et al. 2021a). Adequate arginine–NO signaling is essential for maintaining neurovascular health and cognitive function, while its deficiency has been linked to oxidative stress, neuroinflammation, and impaired neuroplasticity. In murine models, dietary L‐arginine supplementation has demonstrated anti‐stress effects by reducing oxidative stress and improving emotional behavior (Pervin et al. 2021). Moreover, recent clinical evidence suggests that disrupted L‐arginine metabolism contributes to NO deficiency, impaired cerebral perfusion, and heightened neuroimmune burden in critically ill patients, emphasizing the relevance of restoring arginine homeostasis to support neurological recovery (Zinellu et al. 2024). Together, these findings imply that vitamin D3–induced microbial shifts promoting arginine biosynthesis may buffer stress‐induced neuroendocrine dysregulation and enhance behavioral resilience through NO‐mediated mechanisms, providing a plausible link to the partial recovery observed in the CSDVD group.

Additionally, the enrichment of glycolysis‐related pathways, such as Glycolysis I, in the CSDVD group may reflect enhanced microbial support for host energy metabolism under stress. Zhu et al. (2025) demonstrated that maternal vitamin D promotes glycolytic reprogramming of offspring CD4⁺ T cells, enhancing immune tolerance and reducing inflammation, with downstream effects on reducing autism spectrum disorder (ASD)‐like behaviors. This highlights the critical role of metabolic fitness in neuroimmune resilience. In our study, the observed increase in microbial Glycolysis I and II pathway abundance in the CSDVD group may reflect a similar mechanism: Vitamin D3–induced microbial shifts potentially enhance glycolytic capacity, thereby supporting central and peripheral immune recovery under chronic stress. Such metabolic reprogramming may improve energy availability and modulate inflammation, providing a plausible mechanistic link to the behavioral improvements observed in vitamin D3–treated sleep‐disrupted mice. Furthermore, the capacity of vitamin D3 supplementation to modulate microbial energy metabolism pathways may reinforce host mitochondrial function and ATP production, facilitating neuronal and immune recovery. Together, these data suggest a microbiota–immune–behavior axis whereby vitamin D3 shapes microbial metabolic output (e.g., glycolysis), which in turn supports host immune cells and neurobehavioral resilience under sleep–wake disruption.

In the current study, sleep desynchrony significantly reduced the expression of tight junction proteins ZO‐1, occludin, and claudin in the proximal colon, indicating impairment of intestinal barrier function. Vitamin D3 treatment significantly restored the expression levels of ZO‐1 and claudin, suggesting a protective role of vitamin D3 in maintaining intestinal barrier integrity. By contrast, sleep recovery alone did not have significant effects on the levels of these proteins. Studies have indicated that an increase in the abundance of Firmicutes could lead to gut barrier dysfunction. (Filippo et al. 2010; Schippa and Conte 2014). Therefore, researchers have hypothesized that vitamin D3 treatment can modulate the microbiota composition, resulting in higher and lower levels of Bacteroidetes and Firmicutes, respectively, which could help maintain the intestinal barrier integrity. In addition, in vitro and animal studies have indicated that vitamin D signaling enhances epithelial barrier integrity by regulating the expression of various junctional proteins, defensins, and mucins (Vernia et al. 2022). Finally, although no significant differences in LPS levels were identified among the NS, CSD, CSDR, and CSDVD groups in the current study, sleep desynchrony affected the production of SCFAs in all groups. The CSD group exhibited a significant reduction in propionic acid and butyric acid levels, and vitamin D3 treatment significantly restored these levels. One study reported that the abundance of Muribaculaceae was positively correlated with the concentration of propionic acid (Smith et al. 2019; Yang et al. 2023). In our study, Muribaculaceae abundance was significantly reduced in the CSD group but restored to normal levels following vitamin D3 administration, suggesting that vitamin D3 reversed sleep‐induced microbial loss and subsequently enhanced propionic and butanoic acid production.

Vitamin D3 may exert its protective effects through both direct and indirect mechanisms. Directly, activation of the vitamin D receptor (VDR) has been shown to upregulate tight junction proteins such as occludin and claudin, suppress NF‐κB signaling, and reduce intestinal inflammation, thereby enhancing epithelial barrier function independently of microbial changes (Cai et al. 2025). Indirectly, vitamin D3–induced shifts in microbiota composition may promote the expansion of beneficial SCFAs‐producing taxa, which are known to support gut barrier integrity and regulate systemic immune responses (Gao et al. 2023). Notably, SCFAs can also modulate the hypothalamic–pituitary–adrenal (HPA) axis by reducing CRH and cortisol levels, attenuating neuroinflammation, and further preserving epithelial integrity (Wang et al. 2023). In the present study, vitamin D3 supplementation partially restored SCFAs levels, with a trend toward increased propionic acid compared with the CSD group, supporting a potential role in both gut and neuroendocrine regulation. These proposed pathways merit further investigation using microbial depletion, fecal transplantation, or VDR knockout models to establish causal relationships.

The physiological effects of sleep desynchrony or sleep deprivation vary substantially with its duration. Short‐term deprivation typically induces reversible changes in stress responses and neuroplasticity, as shown by Murata et al. (2024) and Popescu et al. (2025), who reported that rebound sleep restored hippocampal function and normalized gene expression patterns. In contrast, CSD becomes a common workstyle among workers and students, leading to more severe and potentially irreversible impairments that often go unnoticed. Tang et al. (2024) and Zheng et al. (2025) found that prolonged sleep loss caused oxidative damage, visual deficits, and reproductive dysfunction, which were not readily reversed by short‐term recovery sleep. These findings suggest that while acute sleep loss may be recoverable, chronic deprivation can result in lasting structural and metabolic damage. Nonetheless, differences in experimental methods, sleep deprivation protocols, and recovery conditions complicate direct comparisons across studies, highlighting the need for standardized models to better characterize the impacts of sleep loss and recovery.

Despite the clear biological trends observed in behavior, immunological markers, and gut microbiota composition, this study is limited by its small sample size (*n* = 6 per group), which may reduce the statistical power to detect subtle differences, especially in highly variable datasets such as the microbiome. This limitation may have contributed to the lack of significant group differences observed in certain outcomes, such as plasma LPS levels and specific inflammatory markers. Another important limitation is the use of only male mice, which may not fully capture potential sex‐specific responses to vitamin D3, especially in mental health and HPA axis regulation. Prior studies suggest that vitamin D metabolism, stress responsivity, and behavioral outcomes can differ between sexes due to hormonal and receptor‐level variations (Bertone‐Johnson 2009). Although our sample size is consistent with standard exploratory animal studies, future research should include larger cohorts and both male and female animals to validate and extend these findings while enhancing translational relevance. Additionally, although acute and chronic sleep deprivation are often distinguished by duration, with acute deprivation typically referring to experimental protocols lasting less than 3 days and chronic deprivation involving sustained or repeated sleep restriction for 3 days or more, the boundary between short‐term and long‐term effects remains difficult to delineate (Alhola and Polo‐Kantola 2007). This ambiguity is partly attributable to methodological heterogeneity across studies, including variations in sleep disruption paradigms, recovery schedules, and behavioral outcome measures.

Nevertheless, this study provides an integrative perspective on the physiological consequences of chronic sleep desynchrony and highlights the potential of vitamin D3 supplementation as a partial corrective intervention. To our knowledge, this is the first animal study to simultaneously evaluate behavioral performance, metabolic status, stress responses, immune parameters, and gut microbial profiles in the context of chronic sleep desynchrony and vitamin D3 intervention. This multidimensional approach not only improves our understanding of the interplay between circadian health and systemic physiology but also supports the translational potential of vitamin D3 in mitigating the health impacts of shift work and related sleep disturbances.

## 15 Conclusions

The findings of the present study signify the adverse effects of chronic sleep desynchrony, which simulates the effects of long‐term shift work, on various physiological, behavioral, and immunological parameters, including its effects in disrupting gut microbiota composition and intestinal barrier integrity. Chronic sleep desynchrony led to imbalances in immune function and gut microbiota, and subsequent vitamin D3 treatment mitigated these effects. Vitamin D3 treatment effectively restored gut microbial balance, improved intestinal barrier function, and enhanced the levels of SCFAs such as propionic and butanoic acids. Additionally, vitamin D3 treatment helped to normalize HPA axis dysfunction and partially restore locomotor function impaired by sleep disruption. Overall, these findings highlight the therapeutic potential of vitamin D3 in restoring physiological homeostasis and protecting against the detrimental effects of sleep desynchrony.

## Author Contributions

**Changwei W. Wu**: conceptualization, project administration, resources, supervision, writing – review and editing. **Yen‐Ju Huang**: resources, supervision, writing – review and editing. **Hsien‐Yu Fan**: methodology, data curation, formal analysis. **Jin‐Wei Xu**: data curation, writing – review and editing. **Jun‐Lan Zeng**: investigation, methodology, validation, visualization. **Yu‐Chen S. H. Yang**: investigation, methodology, validation, visualization. **Yu‐Tang Tung**: conceptualization, data curation, formal analysis, funding acquisition, investigation, methodology, project administration, resources, supervision, validation, writing – review and editing.

## Funding

This work was financially supported by a research grant from the National Science and Technology Council (NSTC112‐2320‐B‐005‐009‐MY3), Ditmanson Medical Foundation Chia‐Yi Christian Hospital (R111‐40), and National Health Research Institutes (NHRI‐EX113‐11129EI).

## Ethics Statement

All experimental procedures involving animals were thoroughly examined and approved by the IACUC of National Chung Hsing University, and the protocol guidelines 111‐004R were approved by the ethics committee of the IACUC. The current study was carried out in compliance with the ARRIVE guidelines.

## Consent

The authors have nothing to report.

## Conflicts of Interest

The authors declare no conflicts of interest.

## Supporting information




**Supplementary Material**: brb371084‐sup‐0001‐SuppMat.docx

## Data Availability

The datasets used and analyzed in this study are available from the corresponding author upon reasonable request.
