# Supplementary material for: Vitamin D3 Improves Hypothalamic–Pituitary–Adrenal Axis Function, Immunological Responses, and Gut Dysbiosis in Sleep Desynchrony
Source: Brain Behav. 2025 Nov 21;15(11):e71084. doi: 10.1002/brb3.71084 (PMC12638446; doi:10.1002/brb3.71084)
Supplement: Supplementary file 1 — Supplementary Material: brb371084‐sup‐0001‐SuppMat.docx [file BRB3-15-e71084-s001.docx]

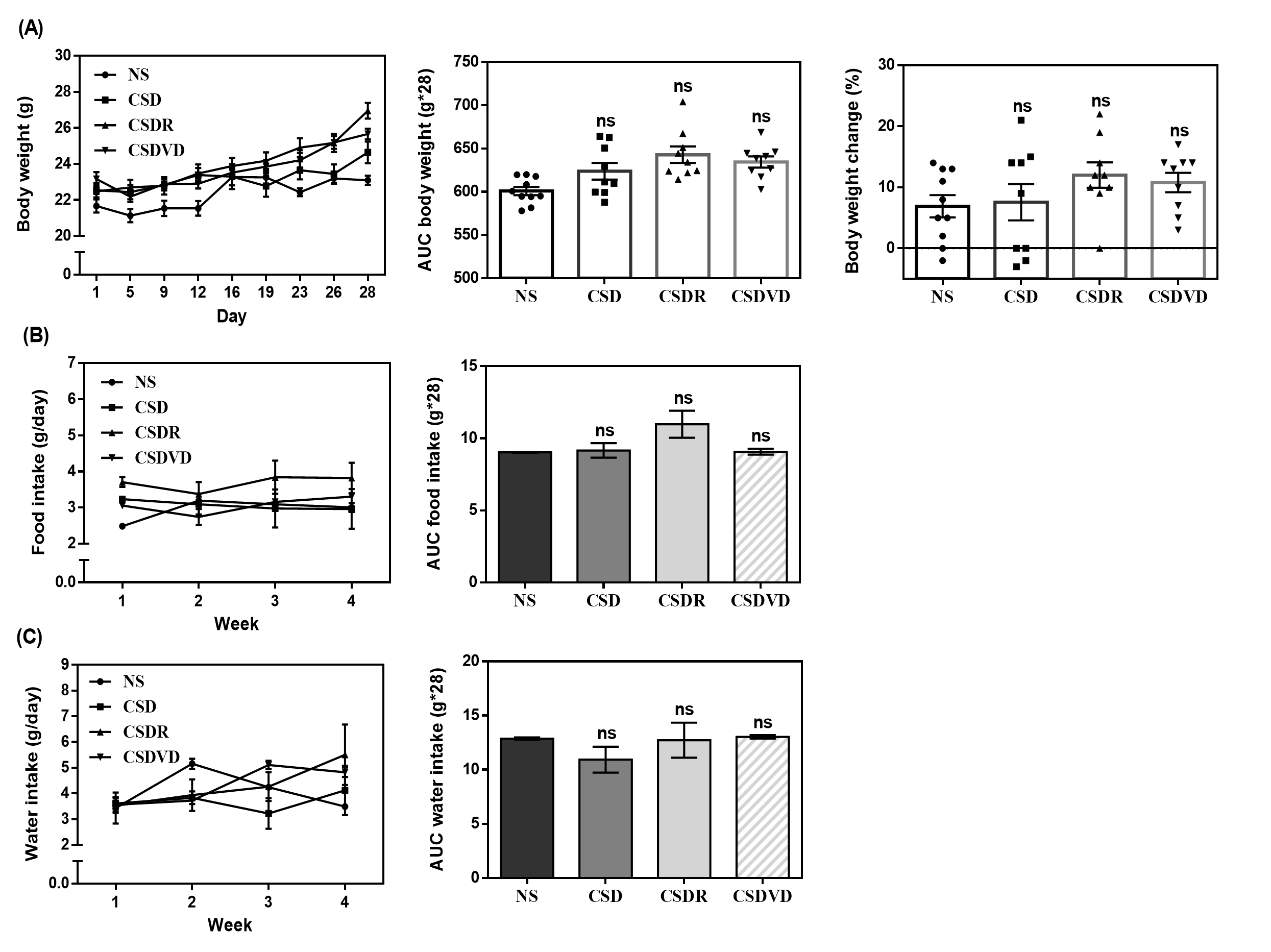
**FIGURE S1.** Effects of vitamin D3 treatment and sleep recovery on body weight, food intake, and water intake in mice with sleep desynchrony. (A) 28-day body weight growth curve, area under the curve (AUC) for 28-day growth curve, and 28-day change in body weight. (B) 28-day food intake and AUC for 28-day food intake. (C) 28-day water intake and AUC for 28-day water intake. NS: Normal sleep group; CSD: Chronic sleep deprivation group; CSDR: CSD followed by sleep recovery; CSDVD: CSD combined with vitamin D3 supplementation. Values are presented as means ± SEMs (*n* = 6). The unpaired one-sided U test was performed for statistical analysis. ns indicates no statistical significance.
